# Transcriptome Level Reveals the Triterpenoid Saponin Biosynthesis Pathway of *Bupleurum falcatum* L.

**DOI:** 10.3390/genes13122237

**Published:** 2022-11-29

**Authors:** Yuchan Li, Jun Zhao, Hua Chen, Yanping Mao, Yuping Yang, Liang Feng, Chuanxin Mo, Lin Huang, Dabin Hou, Ma Yu

**Affiliations:** 1 School of Life Science and Engineering, Southwest University of Science and Technology, 59 Qinglong Road, Mianyang 621010, China; 2 Laboratory of Medicinal Plant Cultivation, Institute of Medicinal Plant Development (IMPLAD), Chinese Academy of Medical Sciences & Peking Union Medical College, Beijing 100193, China

**Keywords:** *Bupleurum falcatum*, transcriptome, saikosaponin biosynthetic pathway, WGCNA, gene expression

## Abstract

*Bupleurum falcatum* L. is frequently used in traditional herbal medicine in Asia. Saikosaponins (SSs) are the main bioactive ingredients of *B. falcatum*, but the biosynthetic pathway of SSs is unclear, and the biosynthesis of species-specific phytometabolites is little known. Here we resolved the transcriptome profiles of *B. falcatum* to identify candidate genes that might be involved in the biosynthesis of SSs. By isoform sequencing (Iso-Seq) analyses of the whole plant, a total of 26.98 Gb of nucleotides were obtained and 124,188 unigenes were identified, and 81,594 unigenes were successfully annotated. A total of 1033 unigenes of 20 families related to the mevalonate (MVA) pathway and methylerythritol phosphate (MEP) pathway of the SS biosynthetic pathway were identified. The WGCNA (weighted gene co-expression network analysis) of these unigenes revealed that only the co-expression module of MEmagenta, which contained 343 unigenes, was highly correlated with the biosynthesis of SSs. Comparing differentially expressed gene analysis and the WGCNA indicated that 130 out of 343 genes of the MEmagenta module exhibited differential expression levels, and genes with the most “hubness” within this module were predicted. Manipulation of these genes might improve the biosynthesis of SSs.

## 1. Introduction

*Radix Bupleuri* (Chai Hu in Chinese) is the dried roots of the genus *Bupleurum*, Apiaceae family, which has been widely used in oriental traditional medicine for more than 2000 years, due to its remarkable therapeutic effect on fever, inflammation, influenza, hepatitis, malaria, and menopausal syndrome [1]. The principal bioactive constituents of *Radix Bupleuri* are its pentacyclic triterpenoid saponins, commonly known as saikosaponins (SSs), which are comprised of oleanane-type and ursane-type glycosides. Over 130 SSs have been isolated to date; according to their diverse skeleton structure, SSs are divided into 14 types [2]. SSs possess diverse pharmacological effects, especially SSa and SSd, which have immunomodulatory, anti-inflammatory, anticancer, antiviral, antibacterial, sedative, analgesic, and other pharmacological effects. Potent antioxidant, hepatoprotective, and cytotoxic effects were also reported [3,4].

Different genotypic varieties of *Bupleurum* plants differ significantly in the content and proportion of SSs [5,6], which are regulated by interior factors, e.g., growth stage, root structure, and environmental conditions, such as drought, light deficiency, and fertility [7,8,9,10,11,12,13]. SSs are mainly in the pericycle and primary phloem in young roots, while in mature roots, they were mainly concentrated in the secondary phloem and vascular cambium [14]. Therefore, combining cultivation techniques and manipulating gene expressions with regard to the biosynthesis of SSs could be a more operative approach to improving the SSs yield.

SSs are synthesized via the isoprenoid pathway, initially, IPP (isopentenyl pyrophosphate) and DMAPP (dimethylallyl diphosphate) are synthesized via the MVA (mevalonate) pathway and MEP (methylerythritol phosphate) pathway, respectively. Then the GPP (geranyl diphosphate) and DMAPP transform into farnesyl pyrophosphate (FPP), which then transforms into 2,3-oxidosqualene via two consecutive enzyme reactions. The β-amyrin synthase (β-AS) is responsible for cyclizing 2,3-oxidosqualene to generate β-amyrin. Finally, cytochrome P450s (CYPs) catalyze a series of hydroxylation/oxidation reactions and UDP-glycosyltransferases (UGTs) catalyze glycosylation reactions, so as to generate various saikosaponin monomers [15,16,17]. Several genes encoding SS biosynthesis enzymes were cloned and identified in *B. kaoi* (Bk), *B. chinense* (Bc), and *B. falcatum* (Bf), such as *BcSE1*, *BfSS1*, *CYP716Y1*, and *BkβAS* [18,19,20,21]. Nevertheless, the biosynthesis of SSs varies among species, and the underlying molecular regulatory mechanisms are still elusive. In the present study, weighted gene co-expression network analysis (WGCNA) was used to analyze the transcriptome and its relation with SSs content, so as to unearth the regulation networks of the SS biosynthesis route of *B. falcatum*.

## 2. Materials and Methods

### 2.1. Sampling of B. falcatum

A *B. falcatum* variety was obtained from a medicinal plant farm (altitude 600 m) in Rongxian in October 2013, which was authenticated by Professor Jianhe Wei (Institute of Medicinal Plant Development, Peking Union Medical College & Chinese Academy of Medical Sciences). The voucher specimen of this genotype (No. 0320130122) was stored in the herbarium of the School of Life Science and Engineering, Southwest University of Science & Technology, Mianyang, China.

Bf seeds were put on humid filter paper before germination. Sprouts were grown in modified Hoagland’s nutrient media. The growth conditions were 24 ± 1 °C, a light/dark cycle of 12/12 h, and 55–65% relative humidity. The 5- and 15-day-old sprouts were subject to isoform sequencing (Iso-Seq), transcriptome scrutiny, and SSa/SSd quantifications. S1 was the whole fresh roots of 5-day-old seedlings; S2 was a 5 mm portion of the 15-day-old seedling root tips without the differentiation region; S3 was a 15-day-old seedling root with removed root tips as previously described (Supplementary Figure S1) [22].

### 2.2. Saikosaponin Extraction and HPLC (High-Performance Liquid Chromatography)

Samples S1–S3 with triplicates of each were freeze-dried for 72 h. The Waters HPLC system (Waters 1525 Binary HPLC Pump, Milford, MA, USA)) and an ASB-vensil C18 column (4.6 mm × 250 mm, 5 µm) were used to determine the SSa and SSd content. Their reference standards were purchased from the National Institutes for Food and Drug Control, Beijing, China. The determination methods and conditions previously described were utilized [23].

### 2.3. PacBio Iso-Seq Library Construction and Transcriptome Analyses

The root and leaf of the *B. falcatum seedling* were blended and subject to Iso-Seq sequencing library creation. The Iso-Seq analysis was performed as previously described [24]; the full-length transcript sequencing was conducted on the PacBio Sequel system (Pacific Biosciences, San Diego, CA, USA) following the isoform sequencing protocol, and the Clontech SMARTer PCR cDNA Synthesis Kit and BluePippin Size Selection System were utilized as described by the manufacturer PacBio (PN 100-092-800-03, San Diego, CA, USA).

The transcriptome sequencing of S1, S2, and S3 was performed on the HiSeq 2500 platform (Illumina, San Diego, CA, USA). Three replications were included in this study [25].

### 2.4. PacBio Sequencing Read Processing

The raw sequencing data were processed on the PacBio Sequel platform and SMRTLink5.1 software was utilized to divide the subread BAM files into non-CCS (circular consensus sequence) and CCS subreads. CCSs were then split into FL (full-length) and non-FL (nFL) reads after identifying the poly (A) tail signal and 5′/3′ adaptors by pbclassify.py script. The FL transcripts were clustered by the ICE (iterative clustering for error correction) algorithm and then treated by arrow polishing. The additional nucleotide errors in consensus reads were proofread using the clean RNA-seq data through the software LoRDEC [26]. Finally, CD-HIT removed any redundancy in the corrected shared reads to obtain the final transcripts [27].

### 2.5. Functional Annotation of Genes

The full-length transcripts were detected via the ANGEL pipeline to obtain the protein-coding sequences (CDSs), amino acid sequences, and untranslated region sequences (UTR). Gene function was annotated by blasting CDSs against databases including NCBI nonredundant protein (Nr), Protein family (Pfam), euKaryotic Orthologous Groups (KOG), and Swiss-Prot, with the threshold *E* ≤ 10^−10^. A BLAST search was also performed against the Nucleotide sequence database (Nt) with the cutoff *E*-value 1 × 10^−10^ [28,29,30,31]. In addition, the KEGG (Kyoto Encyclopedia of Genes and Genomes) pathway and GO (Gene Ontology) functional enrichment analyses were implemented in the GOseq R package (3.6.2) and KOBAS software, respectively [32].

### 2.6. TF Prediction and lncRNA, SSR Analysis

CDSs of isoforms were aligned and assigned to different families based on transcription factors (TFs) prediction using iTAK predictive software [33]. The software PLEK and CNCI were adopted to assess the protein-coding potential of transcripts and distinguish protein-coding and non-coding sequences [34,35]. The CPC method uses biological sequence characteristics to determine the protein-coding likeliness of transcripts by comparing them with protein databases. Finally, an hmmscan homology search was performed via Pfam-scan databases to determine the LncRNA (long non-coding RNA). Simple sequence repeats (SSRs) were identified by MISA (http://pgrc.ipkgatersleben.de/misa/misa.html, accessed on 29 May 2020.), a method that identifies and localizes typical SSRs and composite ones intruded by bases.

### 2.7. Quantification of Gene Expression and Differentially Expressed Genes (DEGs) Analysis

The Bowtie2 program in RSEM removed superfluous sequences to acquire ultimate high-quality transcripts, which were the reference transcriptome. Then the levels of gene expression for each sample were estimated [36]. The clean reads were mapped to the reference transcriptome to obtain read counts compared to each gene in each sample. RSEM provides a method using the Bowtie2 program to calculate gene expression abundances via FPKM (fragments per kilobase per million mapped reads).

The gene readcounts were further analyzed in DESeq2 [37] to identify statistically significant DEGs among three root tissues. Additionally, *p*-values were fine-tuned with the Benjamini and Hochberg approach to control the FDR (false discovery rate) [36]. Genes with an adjusted *p*-value < 0.05 were designated as DEGs. Finally, the mean FPKM values of genes were changed to log2 ones for visualization with the heatmap package of R 3.6.2.

### 2.8. Selecting Candidate Genes of Saikosaponin Biosynthesis

Gene families related to saikosaponin synthesis pathways were selected to form Iso-Seq and transcriptome data, including AACT (acetyl-CoA acetyltransferase), HMGS (hydroxymethylglutaryl-CoA synthase), HMGR (3-hydroxy-3-methylglutaryl-coenzyme A reductase), MK (mevalonate kinase), PMK (phosphomevalonate kinase), MVD (mevalonate pyrophosphate decarboxylase), DXS (1-deoxy-D-xylulose-5-phosphate synthase), DXR (1-deoxy-D-xylulose 5-phosphate reductoisomerase), CMS (2-C-methyl-D-erythritol 4-phosphate cytidylyltransferase), CMK (4-diphosphocytidyl-2-C-methyl-D-erythritol kinase), HDS (4-hydroxy-3-methylbut-2-en-1-yl diphosphate synthase), MCS (2-C-methyl-D-erythritol 2,4-cyclodiphosphate synthase), GPS (geranylgeranyl pyrophosphate synthase), FPS (farnesyl diphosphate synthase), IDS (4-hydroxy-3-methylbut-2-enyl diphosphate reductase), IDI (isopentenyl-diphosphate Delta-isomerase), SS (squalene synthase), SE (squalene epoxidase), β-AS, CYP, and UGT. Herein a total of 1033 unigenes were selected in this work (Supplementary Table S1).

### 2.9. WGCNA

The 1033 candidate genes involved in the SS biosynthesis pathway were the input of R package WGCN, and a weighted co-expression network was built to reveal potential saponin biosynthesis modules of *B. falcatum.* The key parameters were set as follows: soft threshold power, 12; min module size, 20; merge cut height, 0.25. The ME (module eigengene, the first principal component of a module) value was computed for each module, so as to assess the relationship with SSs. The genes in the module which significantly related to saikosaponin content were analyzed in the DESeq package of R, and candidate genes with P_adj_ < 0.05 and log2 (fold change) > 2 selected by DESeq were defined as DEGs. The hub genes of the putative saikosaponin synthesis network were assigned based on the edge number and *k_ME_* values [38]. The top score hub genes were computed and sorted with the MCC method implemented in the Cytoscape plugin cytoHbba [39]

### 2.10. Validation by qRT-PCR

From the hub genes, key enzyme genes that regulate the metabolic flux of MEP and MVA pathways, such as AACT, HMGR, and DXS, and β-AS, CYP, and UGT genes involved in the biosynthesis of SSs were selected. In qRT-PCR, gene-specific primers were used to validate the expression of 10 metabolic genes (Supplementary Table S2). The cDNA of S1, S2, and S3 were the same as those used for sequencing. The qRT-PCR reaction was conducted with a Green qPCR SuperMix kit (Transgen Biotech, Beijing, China) and CFX96 Real-time PCR system (Bio-Rad, Hercules, CA, USA), and the relative expression values were computed with the 2^−ΔΔ^Ct method for each gene, all reactions were carried out in biological triplicate [40,41].

## 3. Results

### 3.1. Saikosaponin a (SSa) and Saikosaponins d (SSd) Content

SSa and SSd were detected with HPLC in each region of the root tissue of *B. falcatum.* The result showed that no peaks of SSa and SSd were identified in the S1 and S2 samples, while they were significantly accumulated in S3 (Table 1). Based on this, the genes following the identical or opposite expression trend among the three samples are worth paying more attention to.

### 3.2. Transcriptome Profile Analysis of B. falcatum

With the aim of profiling the whole picture of transcriptional dynamics during the young root development stage, and identifying genes participating in the SS biosynthesis of *B. falcatum*, the FL transcriptome was obtained by SMRT sequencing of the PacBio Sequel platform. We filtered the raw sequencing data to remove the adaptor and offline sequences less than 50 bp, and finally obtained 10,722,671 subreads based on 26.98G. The filtered subreads were 2517 bp in mean length. Through conditional screening, 485,498 CCSs were obtained (full passes, 1; quality, 0.80). In total, 438,691 FL non-chimeric reads (Flnc), with complete 5′ primers, 3′ primers, and poly-A tails, were acquired; the mean length of the Flnc was 3098 bp.

PacBio, the representative third-generation sequencing technology, possesses long read length superiority but is associated with the disadvantage of a higher single base error rate. Illumina data was therefore employed to correct it to decrease the error rate. After correction, 222,393 consensuses, 4061 N50, and 1962 N90 were obtained. Similar and redundant sequences were deleted with the software CD-HIT, resulting in 222,393 nonredundant transcripts.

The Bowtie2 program in RESM software was applied to align clean reads with unigenes. About 70.04% of clean reads were mapped to the *B. falcatum* reference transcriptome, with approximately 32 million clean reads mapped uniquely to the reference transcriptome assembly for each sample. In total, 124,188 unigenes were assembled.

### 3.3. Functional Annotation of Genes

In the functional annotation of unigenes, the databases Nr, Nt, Pfam, KOG, Swiss-Prot, GO, and KEGG were utilized. The Venn diagram demonstrated that 54,131 genes were concurrently annotated in all these databases (Supplementary Figure S2A). In total, 121,417 genes were annotated in ≥ 1 database, and 119,845 (96.5%) genes were interpreted by the NR database. Based on the sequence homology alignment, 107,815 (86.81%) unigenes were identified against *Daucus carota*; 1011 (0.81%) sequences showed momentous hits for *Vitis vinifera.*

A total of 118,312 genes (95.26%) genes were annotated by the KEGG database, and it revealed that 5915 (4.76%) genes were clustered in the signal transduction, 4564 in the signal transduction pathway, and 3885 (3.13%) in transport and catabolism (Supplementary Figure S2B). A total of 81,594 (65.70%) genes were assigned with GO terms, and the KOG database explained 78,450 (63.17%) genes (Supplementary Figure S2C,D).

### 3.4. Ts Prediction and SSR: LncRNA Analysis

A total of 7256 unigenes were classified into 69 TF families identified from the transcriptome of *B. falcatum* by iTAK software. Among them, the top ten TF families were SNF2, PHD, SET, C3H, WRKY, Jumonji, C2H2, bHLH, B3-ARF, and AP2/ERF-ERF. Moreover, 524 transcripts were predicted as other TFs. Six types of SSR, including dinucleotide, tetranucleotide, hexanucleotide, single nucleotide, pentanucleotide, and trinucleotide repeat sequences, were identified using MISA 1.0 software. LncRNAs are RNA molecules of longer than 200 bp without protein products. In addition, based on 222,393 PacBio Iso-Seq isoforms, CNCI, PLEK, CPC, and Pfam-scan, four tools were used to predict LncRNA, and the predicted number of lncRNAs were 26,648, 17,502, 5875, and 32,894, respectively. The length of lncRNAs was between 200 and 1000 bp, lower than that of mRNAs (1500–3000 bp).

### 3.5. Comparative Analysis of DEGs

**Figure 1:**
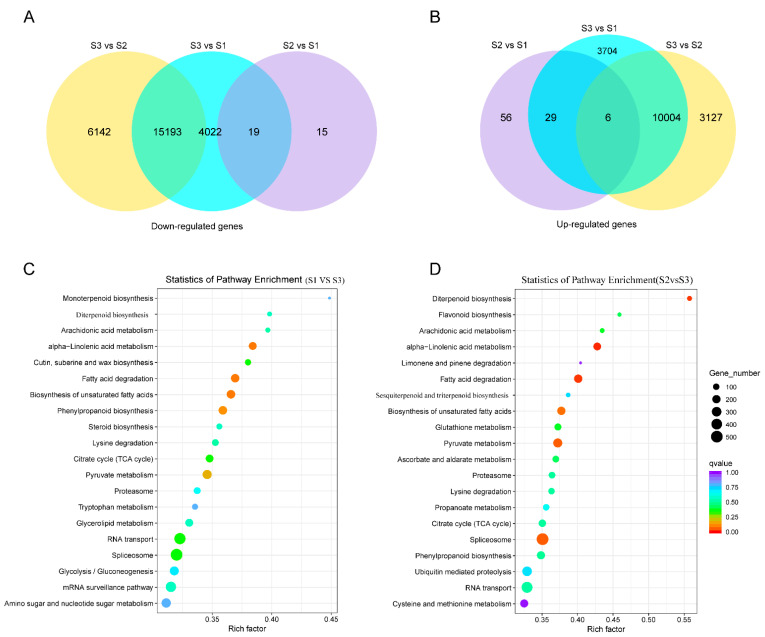
Differentially expressed genes in the seeding root of *B. falcatum* among three samples. (**A**,**B**) Venn diagram of all down-regulated genes and all up-regulated ones from the transcriptome. S1, the whole fresh roots of 5-day-old seedlings; S2, a 5 mm portion of root tips without the differentiation region of 15-day-old sprout; S3, differentiated root tissue of 15-day-old seedlings. (**C**,**D**) Pathway enrichment analysis of DEGs identified in ”S1 vs. S3” (**C**), and “S2 vs. S3” (**D**), based on the KEGG database.

To investigate transcript changes in *B. falcatum* between young root development stages, DEGs were filtered with |log2FoldChange| > 0 & P_adj_ < 0.05. The DEGs numbers of three comparisons (“S1 vs. S2”, “S2 vs. S3”, and “S1 vs. S3”) are shown in Figure 1A,B. Compared to undifferentiated root regions S1 and S2, 15,193 down-regulated and 10,004 up-regulated genes were identified in the taproot differentiation regions of 15-day-old sample S3, respectively. Only 34 or 91 genes were down- or up-regulated in comparison to “S1 vs. S2”. Additionally, in total, 25,197 DEGs genes were identified in S3 in comparison to S1 and S2, and DEGs were annotated in the KEGG pathway (Figure 1C,D). Numerous DEGs involved in specialized metabolism were identified, such as those involved in sesquiterpenoid/triterpenoid production, diterpenoid synthesis, and steroid biosynthesis, among others.

### 3.6. Gene Co-Expression Network Construction

**Figure 2:**
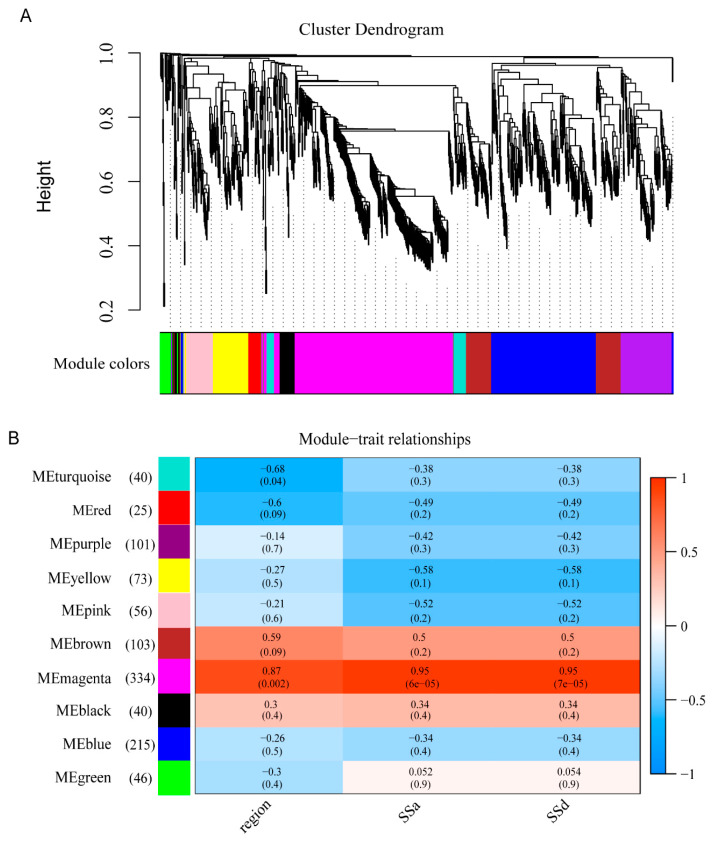
WGCNA. Hierarchical gene clustering was used to sort out the clustering tree of the co-expression network module according to the 1-tom matrix. Each module used a dissimilar color. (**A**) The hierarchical dendrogram displays co-expression modules revealed by WGCNA. One gene is represented by one leaf. Ten modules were found and assigned with different colors based on the calculation of eigengenes. (**B**) Correlation between traits (bottom) and modules (left), with numbers in left brackets showing the gene number of the module. Blue and red represent negative and positive correlations, respectively, and coefficient values are shown. The darker the color, the higher the correlation coefficient. The *p*-values are in brackets.

By pairwise correlation analysis with gene expressions, WGCNA was employed to identify the candidate genes that were highly associated with SSs content in the *B. falcatum.* Ten gene modules of the co-expression network, labeled black, blue, brown, green, magenta, pink, purple, red, turquoise, and yellow, were identified. In each module, the number of target genes was between 25 and 343. At the *p*-value < 0.05 level, the correlation coefficients value among SSs content, different root region, and modules eigengenes revealed that the MEmengta module (343 unigenes) was positively significantly associated with SSa, SSd, and root region tissues (R = 0.95, 0.95, 0.87) (Figure 2).

### 3.7. DEGs in MEmagenta Module Involved in the Saikosaponin Biosynthesis

**Figure 3:**
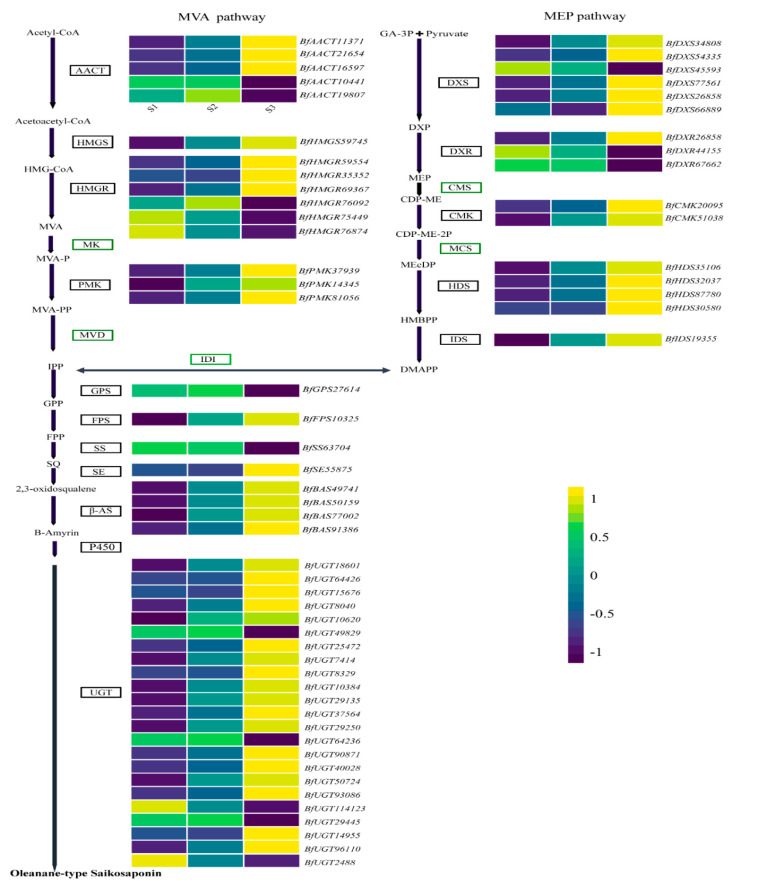
Differential expression of candidate genes of saikosaponin biosynthesis route among S1, S2, and S3. HMG-CoA: 3-hydroxy-3-methy-lglutaryl-COA; MVA-P: 5-phosphoevalonate; MVA-PP: 5-pyrophosphoevalonate; DXP: 1-deoxyxylulose 5-phosphateuvate; CDP-ME: methylerythritol cytidyl diphosphate; CDP-ME-2P: 4-diphosphocytidyl-2-C-methyl-D-erythritol; MEcDP: 2-C-methyl-D-erythritol-2,4-cyclodiphosphate; HMBPP: 4-hydroxy-3-methyl-butenyl-1-diphosphate.

**Figure 4:**
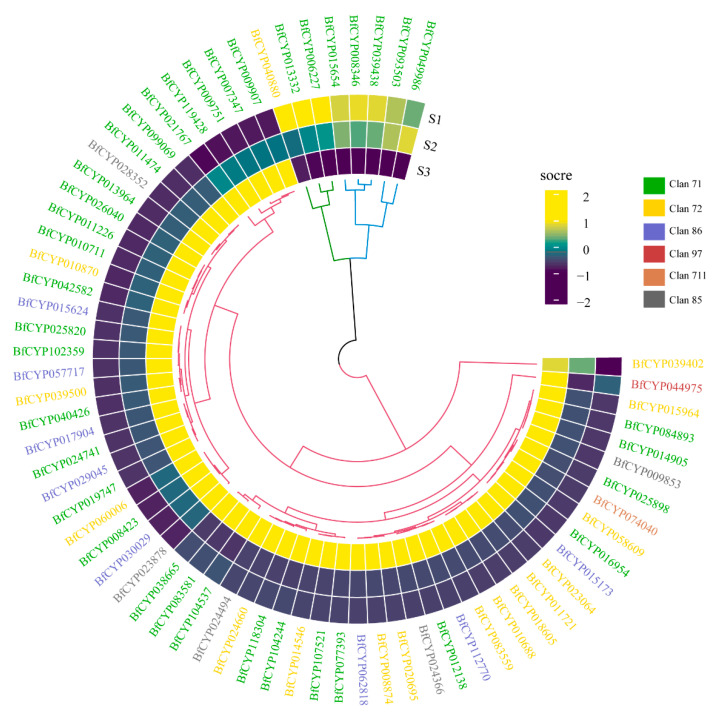
Gene expression profile and cluster analysis of candidate P450. These genes displayed meaningfully different expressions in S3 of *B. falcatum.*

To find genes participating in the differential metabolism of saikosaponins in the MEmagenta module, the expression levels and forms of all transcripts were analyzed in terms of the FPKM transcriptome data. A stringent threshold |log2 Fold change| >1 and P_adj_ < 0.05 screened out DEGs. In total, 130 unigenes closely related to saikosaponin biosynthesis showed significantly different expressions in different root tissue of *B. falcatum* (Supplementary Table S3). Among these DEGs, fifteen genes were divided into MVA pathways, including five AACT genes, one HMGS gene, six HMGR genes, and three PMK genes. For the MEP pathway, five gene families were identified, including DXS (6 unigenes), DXR (3 unigenes), CMK (2 unigenes), HDS (7 unigenes), and IDS (1 unigenes). Additionally, we did not detect DEG in the gene families of MK, MVD, CMS, and MCS. These DEGs, including SS (1 unigenes), SE (1 unigene), β-AS (4), P450s (68), and UGT (23), may convert oleanane-type SSs (Figure 3 and Figure 4).

### 3.8. Hub Gene Selection for the MEmagenta Module

**Figure 5:**
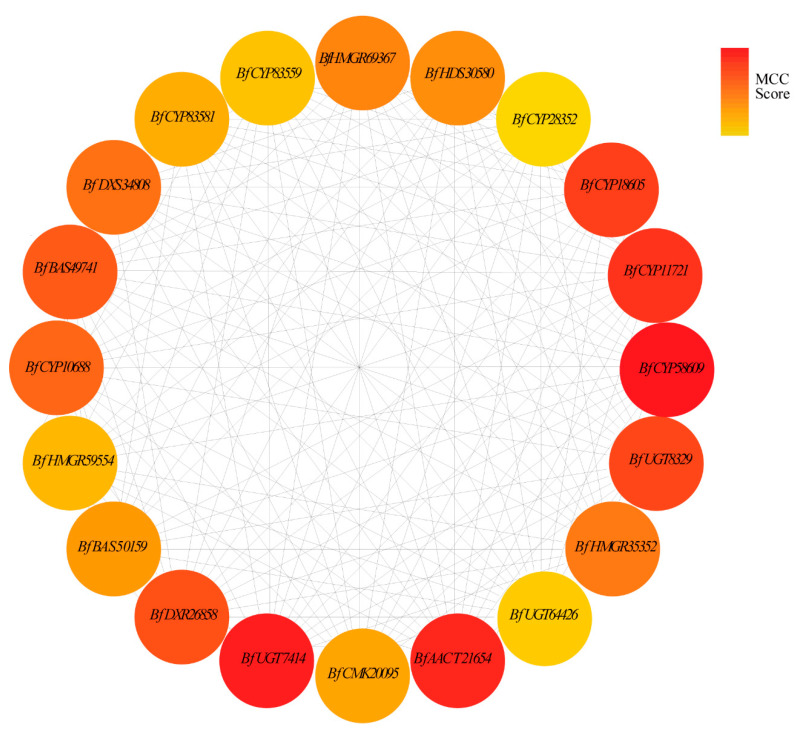
Hub genes of putative triterpenoid biosynthesis pathway. The MCC (maximal clique centrality) algorithm identified hub genes in PPI networks. The red node represents the high MCC score gene, and the yellow represents the low score gene.

Hub genes correlated with differential SSa and SSd levels in root tissues were identified based on the importance of genes in the MEmatenga module. The top 20 genes with the highest MCC values might connect more closely with other genes and play vital roles in the co-expression network (Supplementary Table S4). As shown in Figure 5, the hub gene included AACT (1 unigene), DXR (1 unigene), DXS (1 unigene), HMGR (3 unigenes), HDS (1 unigene), CMK (1 unigene), UGT (3 unigenes), P450 (7 unigenes), and β-AS (2 unigenes).

### 3.9. qRT-PCR

**Figure 6:**
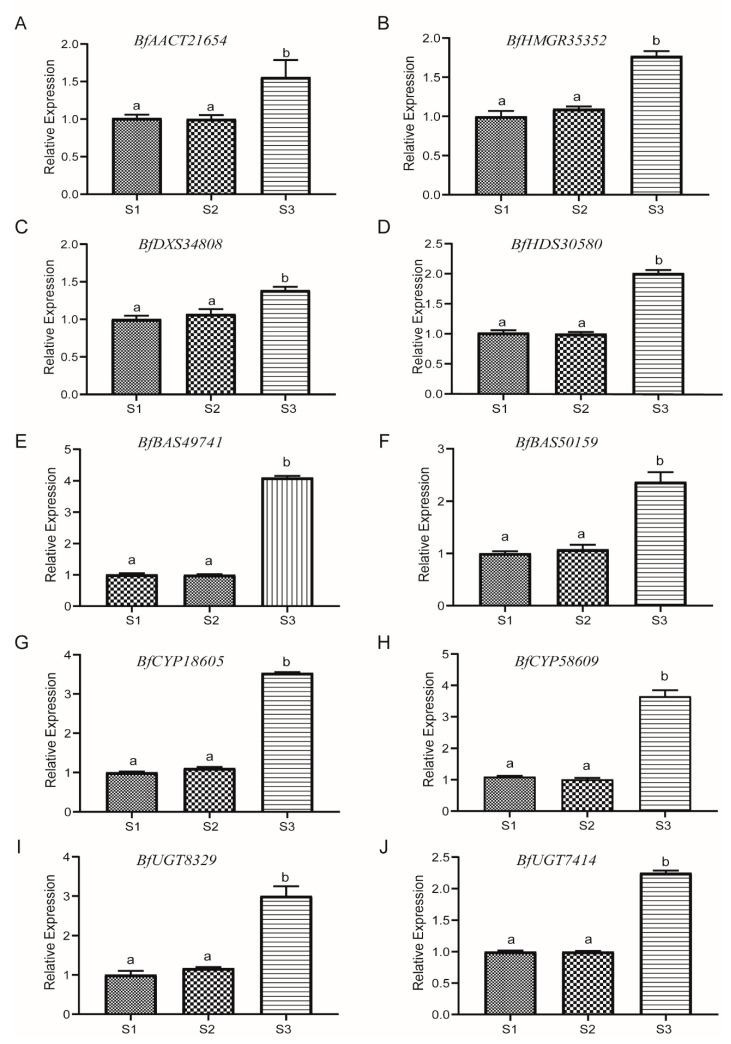
qRT-PCR verification of key genes of saikosaponin biosynthetic pathway. (**A**–**J**) The relative expression patterns of *BfAACT21654, BfHMGR35352, BfDXS34808, BfHDS30580, BfBAS49741, BfBAS50159, BfCYP18605, BfCYP58609, BfUGT8329* and *BfUGT7414*. The bar represents the standard error (SE). Data are presented as mean ± SE. Different letters represent different levels of significant difference.

qRT-PCR was conducted to confirm the transcriptome data and validate the hub genes expression level. The following genes were tested with triplicates, i.e., *BfAACT21654*, *BfHMGR35352*, *BfDXS34808*, *BfHDS30580*, *BfBAS49741*, *BfBAS50159*, *BfCYP18605*, *BfCYP58609*, *BfUGT8329* and *BfUGT7414*. The qRT-PCR showed that all genes had a higher transcript level in mature root tissue when compared with other parts of the root meristem. Therefore, SSs highly produced in mature root tissue may be caused by these highly expressed genes. These results agreed with transcriptome data (Figure 6).

## 4. Discussion

In the present study, we first reported the overall status of the full-length transcriptome profile of *B. falcatum* and identified 1033 unigenes of 20 families involving SSs synthesis pathways from Iso-Seq and transcriptome data. The WGCNA analysis of those 1033 unigenes revealed that only the co-expression module of MEmagenta, which contained 343 unigenes, was highly correlated with SSa and SSd. Additionally, 130 out of 343 genes of the MEmagenta module exhibited differential expression levels. We also identified 20 hub genes with the highest correlation in this module. Furthermore, real-time quantitative results also confirmed that these hub genes increased significantly in mature root tissue (S3).

MEP/MVA pathways participate in the biosynthesis of triterpenoid backbones in planta [42]. AACT catalyzes the formation of acetoacetyl-CoA via transferring an acetyl group from one acetyl-CoA to another, the first step of the MVA route; it plays a fundamental role in triterpenoid biosynthesis. For instance, overexpression of *GL-AACT* promoted the buildup of triterpenoids in *Ganoderma lucidum* [43]. As the first rate-limiting enzyme of the MVA route, HMGR regulates the terpenoid metabolism; for instance, Lee et al. [44] manipulated the HMGR gene by constructing biotin carboxyl carrier protein for co-expression in *Nicotiana benthamiana*, which increased the yield of sesquiterpenoids and triterpenoids. DXS is a limiting enzyme catalyzing the initial step of the MEP route in plant isoprenoid biosynthesis, just like HMGR in the MVA pathway [45]. In addition, reestablishing natural product pathways based on the plant as a chassis, deregulating crucial enzymes in the MVA or MEP pathway, and increasing the production of natural active substances are currently common approaches to increase the abundance of target metabolites, such as the production of taxadiene 5α-hydroxytaxadiene engineered by MEP pathway, which achieved 56.6 μg/g and 1.3 μg/g, respectively [46]. The enhanced production of artemisinic acid via the engineered MVA route attained an almost 500-fold yield [47]. In our study, five AACT genes, six HMGR genes, and six DXS genes were identified. Among them, three AACT genes, three HMGR genes, and five DXS genes showed significantly up-regulated expression in S3, with the exception of two AACT genes, three HMGR, and one DXS gene, which showed a conversely expressional tendency. Therefore, manipulating these genes with the same expression trend as SSs content in the root of *B. falcatum* could improve the availability of the precursor (2,3-oxidosqualene) for biosynthesizing triterpenes, especially hub genes, such as *BfAACT21654*, *BfDXS34808*, and *BfHMGR35352* (Figure 6A–C).

The C5 unit IPP generated by MEP/MVA routes can be transformed into the isomer DMAPP via IDI [48]. However, we failed to identify the expression disparity of the IDI gene. IPP/DMAPP are transformed into FPP and GPP via FPS and GPS, respectively. The *BfGPS27614* transcript expression decreased in S3 of *B. falcatum*, whereas the expression of the FPS gene *BfFPS10325* increased. A previous study revealed that when *BfSS1* was knocked down in transgenic Bf, the transcript buildup increased in downstream genes, e.g., SE and cycloartenol synthase. In this study, *BfSS63704* was down-regulated in S3, while *BfSE55875* was up-regulated, which showed a similar result to the study by Kim et al. (2011) [21].

β-AS is a member of the OSC (oxidosqualene cyclase) family, which acts in the initial step of the biosynthesis of SSs, and the resultant β-amyrin is the backbone of various SS monomers [49]. β-AS was an important branch point between specialized and primary metabolism and could regulate the metabolic accumulation of triterpenoid saponins. There is a positive correlation between β-AS activity and triterpene saponin content in plants. The down-regulation of β-AS gene expression by RNA interference in *Panax ginseng* hairy roots led to reduced β-amyrin and oleanane-type ginsenoside [50]. In *Medicago truncatula*, β-AS ectopic expression led to an increased yield of triterpene saponins [51]. Similarly, in our study, four β-AS genes were identified by WGCNA to be closely related to SSa and SSd. All these β-AS genes were highly expressed in S3, especially *BfBAS49741* and *BfBAS50159* (Figure 6E,F).

The cyclic backbone synthesized by β-AS is subject to site-specific oxidation via CYPs to yield diversified sapogenins, e.g., hydroxylation, carboxylation, and esterification [52,53]. CYP families of land plants were categorized into 11 clans, and the CYP71, CYP51 CYP72, and CYP85 clans are responsible for the biosynthesis of triterpenoids [54]. In addition, the CYP72A family forms the CYP72 clan, and the CYP716 family from the CYP85 clan is primarily involved in pentacyclic triterpenoid oleanolic acid-derived saponin biosynthesis [55,56]. For example, the CY*P716Y1* of Bf was responsible for the C-16α hydroxylation of oleanane triterpenes [18]. In the present study, we identified 68 CYP DEGs that are members of 19 subfamilies of 6 CYP clans (Supplementary Table S5). Among these genes, three *CYP716Y1* genes belonging to the CYP85 clan and nine unigenes belonging to the CYP72 clan showed significant up-regulation in S3. Furthermore, based on the hub gene analysis and RT-PCR results (Figure 5 and Figure 6G,H), it was further confirmed that *BfCYP20695* and *BfCYP58609* may be critical in the biosynthesis of pentacyclic triterpenoid oleanolic acid-derived saponins.

Triterpenoids are further diversified via the scaffold modification of triterpenoids; UGTs catalyze the glycosylation of hydroxylated β-amyrin at C3 [15,57]. In transgenic *P. quinquefolius* hairy roots, *Pq3-O-UGT1* overexpression increased the level of protopanaxadiol group ginsenosides [58]. In this study, we identified 22 UGT genes, among these 18 unigenes were significantly up-regulated in S3, especially the hub gene *BfUGT8329*, while four genes exhibited a significant opposite trend. In this study, we identified 23 UGT genes, in addition to 5 genes that showed a down-regulation trend in S3, 18 other single genes showed significant up-regulation, especially the hub gene *BfUGT8329*.

## 5. Conclusions

In conclusion, the present study for the first time reports the full-length transcriptome profile of *B. falcatum*, and we performed a WGCNA and screened 129 DEGs involved in the SS biosynthetic pathway. Based on the up-regulated genes in S3 and identified hub genes, we constructed a hypothetical scheme of the SS biosynthesis pathway (Supplementary Figure S3). Finding the key enzyme genes of the biosynthesis route of the *Bupleurum* genus offers the option for acquiring appreciated triterpene saponins by synthetic biology. Consequently, it is reasonable to identify, clone, and functionally analyze the key enzymes of *B. falcatum* to promote biological research and commercial application of SSs. Moreover, all recognized key enzymes could be useful for diverse breeding studies of *B. falcatum* and other relevant species.

## Figures and Tables

**Table 1:** Contents of SSa and SSd in the young root of Bf.

Sample	Ssa Content (µg/g)	Ssd Content (µg/g)
S1	0	0
S2	0	0
S3	444.56	312.29

## Data Availability

Data are contained within the article or supplementary material.

## Supplementary Materials

The following supporting information can be downloaded at: https://www.mdpi.com/article/10.3390/genes13122237/s1, Table S1: Annotion of candidate genes for saikosaponin biosynthesis in *B. falcatum*; Table S2: Primer sequences used for qRT-PCR validation; Table S3: FPKM values of DEGs in MEmagenta module involved in the saikosaponin biosynthesis; Table S4: Top 20 in network CytoscapeInput-network.txt ranked by MCC method; Table S5: Summary of clan and subfamliy of P450 DEGs in MEmagenta module involved in the saikosaponin biosynthesis; Figure S1: Sampling positions of S1, S2 and S3 at different seedling stages; Figure S2: Functional annotation and classification of *B. falcatum* transcriptome; Figure S3: The hypothetical synthesis pathway for SSs in *B. falcatum*.
