# Supplementary material for: Transcriptome Level Reveals the Triterpenoid Saponin Biosynthesis Pathway of Bupleurum falcatum L
Source: Genes (Basel). 2022 Nov 29;13(12):2237. doi: 10.3390/genes13122237 (PMC9777608; doi:10.3390/genes13122237)
Supplement: Supplementary file 1 [file genes-13-02237-s001.zip › Supplemental_figures_Genes.pdf]

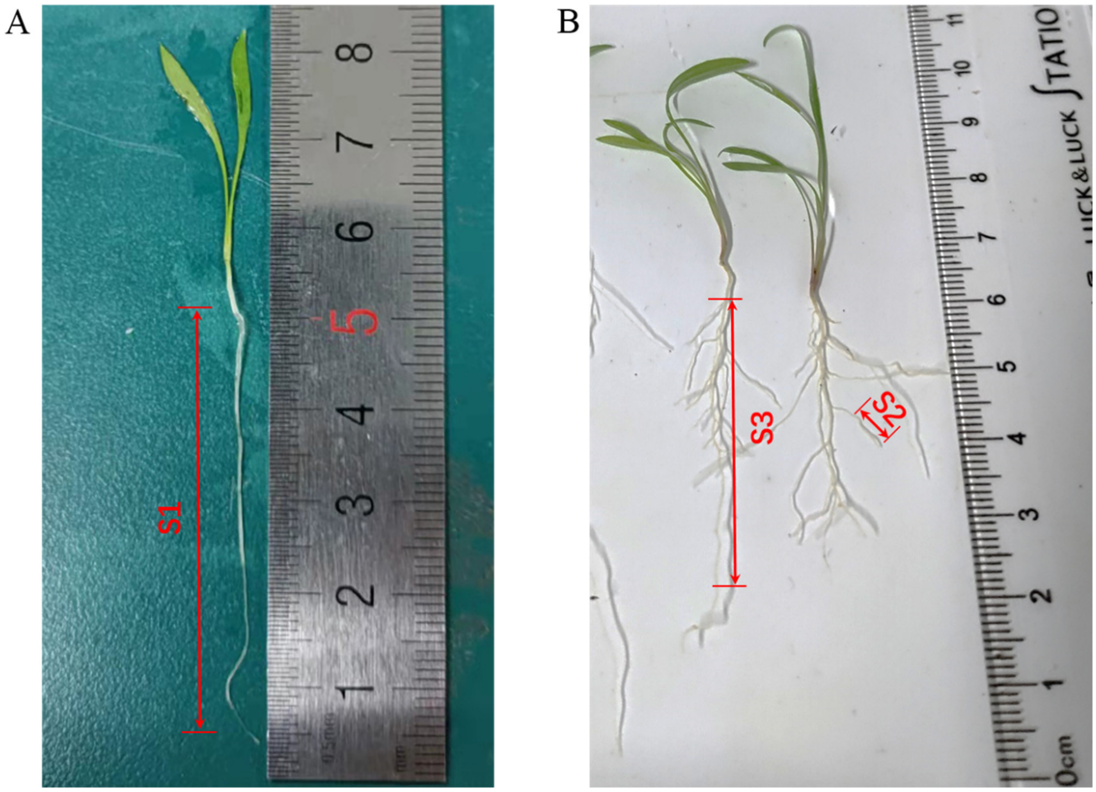

Supplemental Figure S1. Sampling positions of S1, S2 and S3 at different seedling stages. (A) 5-day-old seedlings. (B) 15-day-old seedlings. S1, whole fresh roots of 5-day-old seedlings; S2, 5 mm of the15-day-old seedling root tips without the region of differentiation; S3, 15-day-old seedling root removed the root tips.

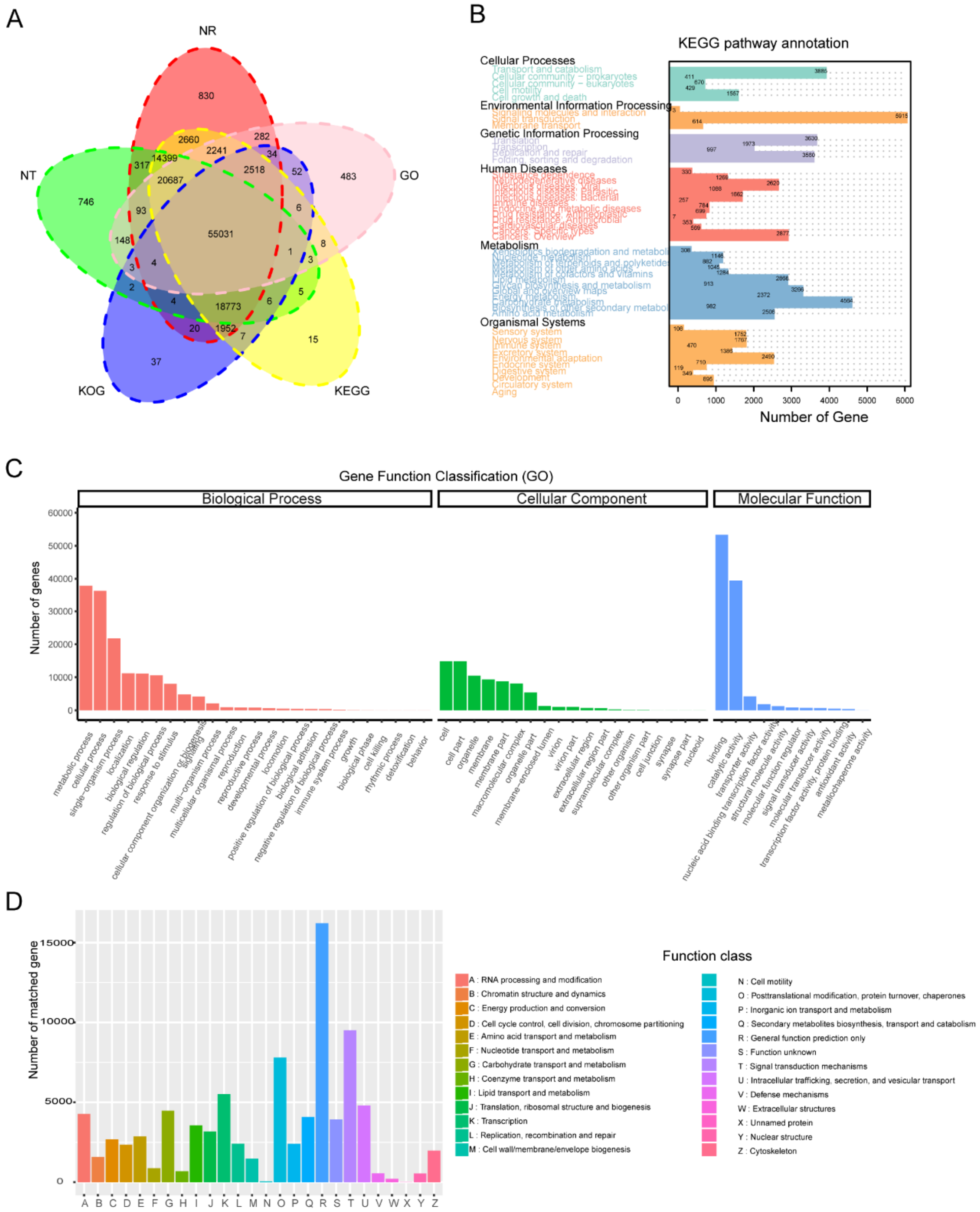

Supplemental Figure S2. Functional annotation and classification of B. falcatum transcriptome. (A)Venn diagram of all isoforms from the transcriptome hits of KOG, non-redundant proteins (NR), Kyoto Encyclopedia of Genes and Genomes (KEGG), and Swiss Prot databases. (B) KEGG classification of B. falcatum transcriptome. (C) Gene ontology classification of *B. falcatum* transcriptome. (D) KOG classification of B. falcatum transcriptome.

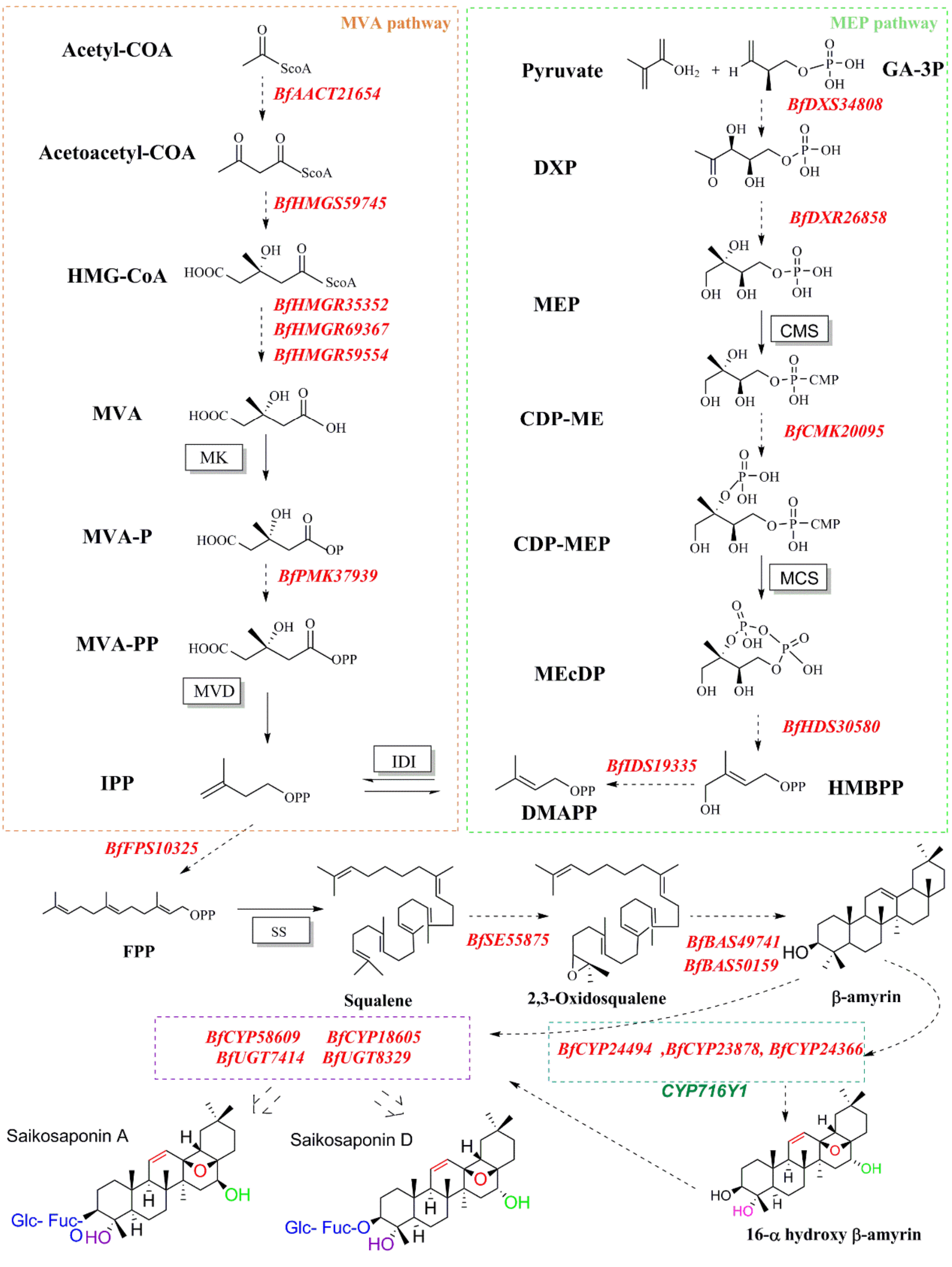

Supplemental Figure S3. The hypothetical synthesis pathway for SSs in *B. falcatum*. AACT, acetyl-CoA acetyltransferase; HMGS, hydroxymethylglutaryl-CoA synthase; HMG-CoA, 3-hydroxy-3-methy-lglutaryl-COA;HMGR, HMG-CoA reductase; MVA, Mevalonic acid; MK, mevalonate kinase; MVA-P, 5-phosphoevalonate; PMK, phosphomevalonate kinase; MVA-PP, 5-pyrophosphoevalonate; MVD, mevalonate pyrophosphate decarboxylase; IPP, isopentenyl pyrophosphomevalonate; DXS, 1-deoxy- D -xylulose-5-phosphate synthase; DXP, 1-deoxyxylulose 5-phosphateuvate; DXR, 1-deoxy- D -xylulose 5-phosphate reductoisomerase; MEP, Methyl erythritol phosphate; CMS, 2-C-methyl-D-erythritol

4-phosphate cytidyltransferase; CDP-ME, Methylerythritol cytidyl diphosphate; CMK, 4-diphosphocytidyl-2-C-methyl-D-erythritol kinase;CDP-ME-2P, 4-diphosphocytidyl-2-C-methyl-D-erythritol; MCS, 2-C-methyl-D-erythritol 2,4-cyclodiphosphate synthase; MEcDP, 2-C-methyl-D-erythritol-2,4-cyclodiphosphate; HDS, (E)-4-hydroxy-3-methylbut-2-enyl diphosphate synthase; HMBPP, 4-hydroxy-3-methyl-butenyl-1-diphosphate; IDS, isopentenyl/dimethylallyl diphosphate synthase; DMAPP, Dimethylallyl diphosphate; IDI, isopentenyl-diphosphate delta-isomerase; GPS, 4-diphosphocytidyl-2-C-methyl-D-erythritol Geranylgeranyl pyrophosphate synthase; FPP, farnesyl pyrophosphate; FPS, farnesyl diphosphate synthase; SS, squalene synthase; SE, squalene epoxidase; β-AS, β-amyrin synthase; P450, cytochromes P450; UGT, uridine diphosphate glycosyltransferases.
